# Early development of turn-taking with parents shapes vocal acoustics in infant marmoset monkeys

**DOI:** 10.1098/rstb.2015.0370

**Published:** 2016-05-05

**Authors:** Daniel Y. Takahashi, Alicia R. Fenley, Asif A. Ghazanfar

**Affiliations:** 1 Princeton Neuroscience Institute, Princeton University, Princeton, NJ 08544, USA; 2 Department of Psychology, Princeton University, Princeton, NJ 08544, USA; 3 Department of Ecology and Evolutionary Biology, Princeton University, Princeton, NJ 08544, USA

**Keywords:** vocal learning, mother–infant, speech evolution, language evolution, communicative pragmatics, primate vocal development

## Abstract

In humans, vocal turn-taking is a ubiquitous form of social interaction. It is a communication system that exhibits the properties of a dynamical system: two individuals become coupled to each other via acoustic exchanges and mutually affect each other. Human turn-taking develops during the first year of life. We investigated the development of vocal turn-taking in infant marmoset monkeys, a New World species whose adult vocal behaviour exhibits the same universal features of human turn-taking. We find that marmoset infants undergo the same trajectory of change for vocal turn-taking as humans, and do so during the same life-history stage. Our data show that turn-taking by marmoset infants depends on the development of self-monitoring, and that contingent parental calls elicit more mature-sounding calls from infants. As in humans, there was no evidence that parental feedback affects the rate of turn-taking maturation. We conclude that vocal turn-taking by marmoset monkeys and humans is an instance of convergent evolution, possibly as a result of pressures on both species to adopt a cooperative breeding strategy and increase volubility.

## 1. Introduction

Social interactions consist of the coupling between two individuals. This coupling represents an autonomous, self-sustaining organization whereby the individuals mutually affect each other [1–3], and, in essence, the coupling takes on a life of its own [3,4]. The bulk of our social interactions take the form of conversations and these are mediated by a turn-taking system. This system is largely universal (present in every language system studied thus far) and organizes speech exchanges efficiently: individuals minimize overlapping the other speaker (by avoiding simultaneous talk) and keep gaps between utterances short [5,6].

The central importance of vocal turn-taking in everyday social interactions and its universality across languages begs the question of both its developmental and evolutionary origins. Developmentally, the ability to participate in shared discourse is fundamental to the establishment of carer–infant bonding. This ability, however, is not present at birth but rather emerges during the first year of life. During the first months of early postnatal life, infant vocal interactions with carers are marked by frequent overlaps [7–9]; turn-taking with minimal overlaps is only achieved by approximately 8–12 months of age [8–11]. Importantly, the development of vocal turn-taking not only serves to establish social bonds, but also facilitates the development of more adult-like vocalizations in infants [12–15]. It is also noteworthy that, in spite of carer influences on infant vocal acoustics, there is no evidence that the development of turn-taking itself is influenced by greater or lesser rates of responses from carers.

How such a developmental system for vocal turn-taking evolved is an important question, as turn-taking may very well be the foundation of more complex linguistics skills [16,17]. Because brains and behaviours do not fossilize, an understanding of the origins of turn-taking must resort to the comparative method. This approach compares the species-typical behaviours of extant primates and humans. With any behaviour that two closely related species share, it can be inferred that their last common ancestor also exhibited that behaviour. In this manner, the comparative method can uncover the turn-taking capacities of extinct ancestors and identify behavioural homologies or products of convergent evolution. However, we must also consider that turn-taking is not only the product of phylogenetic processes, but also ontogenetic ones. As evolution acts on developmental processes to produce adult phenotypes, it is only by comparing developmental trajectories that one can reliably infer similarities or differences across species [18–20]. Importantly, this approach also establishes which non-human species can act as the most effective model systems for human communication and its disorders.

To date, there is no evidence that apes or other Old World primates exhibit vocal turn-taking (beyond call-and-response behaviour) [16] or that the acoustic properties of their vocalizations change to any great degree during infancy or later [21]. Thus, there appears to be no extant primate species that exhibit homologies to human vocal turn-taking and vocal development more generally. For this reason, we have been using marmoset monkeys—a New World species (*Callithrix jacchus*)—to test the hypotheses that vocal turn-taking potentially evolved much earlier than gestural communication in apes, that it does not require ape- or human-specific cognitive capacities or encephalization [16], and that it represents an instance of convergent evolution. We have found that marmosets have a complex system of vocal communication that includes vocal turn-taking—the repeated exchanges of vocalizations between any two individuals for an extended period of time (that is, not simply a call-and-response behaviour among mates or competitors) [16,22,23]. This turn-taking behaviour has the same universal features of human conversational turn-taking (albeit on a different timescale) and exhibits features of a coupled oscillator system [22]. Moreover, marmosets exchange these vocalizations in a cooperative manner, adjusting the amplitude of their voice depending on the distance between individuals [24]. Developmentally, marmoset monkeys also go through a babbling stage [25,26], and the maturation rate of infant vocalizations is influenced by feedback from parents [26]. In this study, we investigated how turn-taking develops in infant marmoset monkeys by measuring their interactions with parents over their first two months of postnatal life. We find that vocal turn-taking development in marmoset monkeys is strikingly similar to that of humans in a number of ways.

## 2. Results

**Figure 1.**
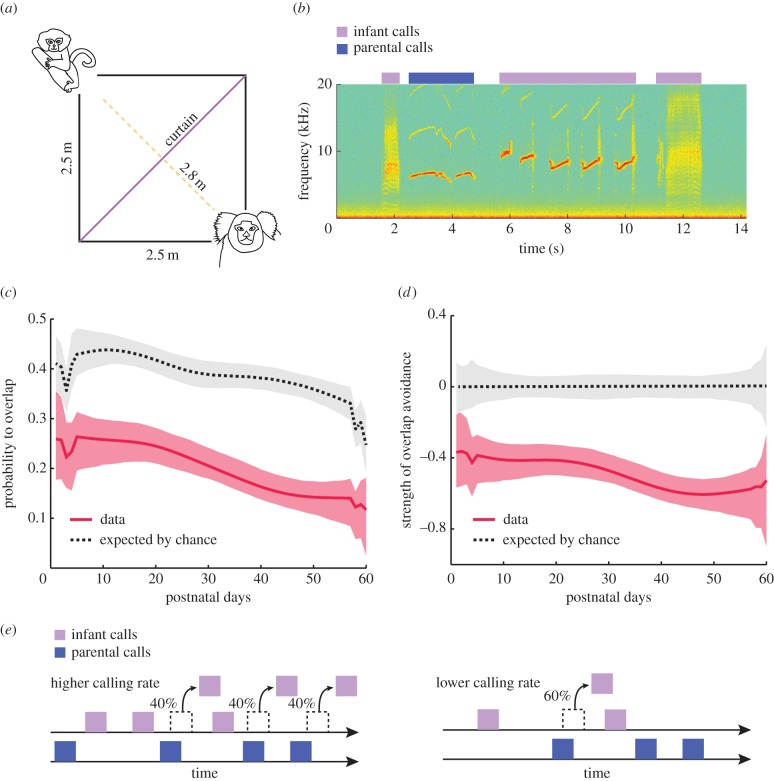
Infant marmosets gradually avoid overlaps over the first two months of life. (*a*) Schematic of the experimental set-up. (*b*) Spectrogram showing an example of vocal interaction between infant and parent marmosets. Warmer colours indicate larger power. The blue rectangle indicates the time segment with a parental call and the purple rectangles indicate time segments with infant calls. (*c*) Development of the probability that an infant call overlaps with an adult call (number of overlaps divided by total number of parental calls). The red line indicates the average probability to overlap and the red shaded region indicates the 95% confidence interval. The black dotted line represents the expected probability of overlap by chance. The grey shaded region represents the 95% confidence interval for the expected probability. (*d*) Development of the strength of overlap avoidance. The red line indicates the average strength of overlap avoidance and the red shaded region indicates the 95% confidence interval. The grey-shaded region indicates the significance at 5% level for the null hypothesis of equality between the data and the expected probability. (*e*) Illustration of the possible mechanism of overlap avoidance. Infant calls are produced according to the spontaneous vocalization dynamics until the occurrence of an overlap.

We recorded vocalizations produced by 10 infant marmosets in a directed context where infants have auditory, but not visual, contact with either their mother or father (figure 1*a*). Two adults placed in this directed context will routinely exchange contact ‘phee’ vocalizations with one another and abide the ‘no-overlap’ rule of turn-taking [22]. In this experimental context, we could thus test if marmoset infants were born with this ability or if, like human infants, it develops over the course of postnatal life. We sampled infant vocal interactions with parents (figure 1*b*) during their first two postnatal months, approximately twice weekly, starting on postnatal day 1 (P1) [26]. Such early and dense sampling is necessary to accurately capture developmental changes in marmosets during this period, as marmoset monkeys develop approximately 12 times faster than humans [27,28]. For example, a one-month old marmoset infant is equivalent to a 1-year old human infant in terms of physical development.

### (a) Infant turn-taking develops during the postnatal period

Figure 1*c* shows that the probability that an infant call overlaps an adult call is approximately 25% at P1 and decreases to approximately 15% after two months (*p* < 0.001, *t*-test for the significance of slope of robust linear regression). One possibility is that it is a simple consequence of a decrease in the rate of call production. If marmoset infants produce fewer calls as they get older, then it could lead to a decrease in overlap. To take this possibility into account, we calculated, using resampling, the expected probability to overlap by chance. To make this calculation, we randomized the timing of both infant and parent vocalizations in each session, and then calculated the probability of infant call overlap. Throughout the first 2 postnatal months, the probability to overlap is smaller than the expected probability due to decreasing call rates (figure 1*c*, dashed black line). Thus, infants actively avoid interrupting parental calls as they get older. However, the decrease in overlap follows the general trend of the decrease in the expected probability to overlap by chance. This shows that the probability to overlap is partly dependent on the rate of call production.

To account for the changing call rates over development, we calculated the strength of overlap avoidance (figure 1*d*). This is the proportion of the expected overlaps that would happen by chance that is avoided by the infant ([probability to overlap − expected probability]/[expected probability]). The strength of avoidance is significantly different from zero throughout development (figure 1*d*, shaded grey region indicates the significance level at 5%), and its value is approximately −0.4 during the first month and becomes −0.6 during the second month. This result suggests that the infant actively avoids overlaps approximately 40% of the time in the first month and approximately 60% of the time after the first month (figure 1*e*).

### (b) Self-monitoring: infants gradually get better at recognizing their own calls

**Figure 2.**
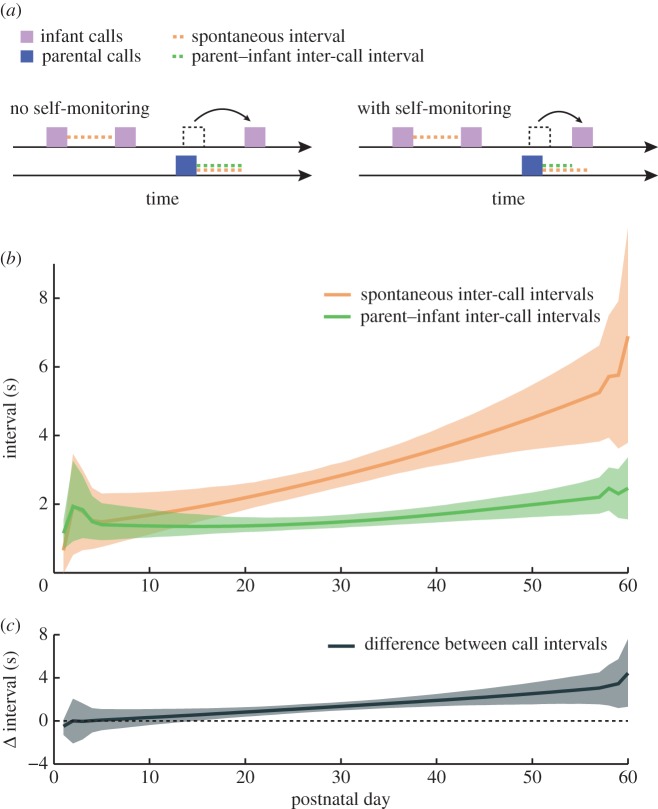
Infant marmoset's self-monitoring capacity develops during the first two months. (*a*) Illustration of the possible ways that infant marmosets can avoid overlaps. They can either reset the timing of vocal production, in which case there is no self-monitoring, or they can respond in a shorter time interval than the interval expected by spontaneous calls, in which case there is self-monitoring. The purple and blue boxes represent infant and parental calls, respectively. The orange and green dotted lines indicate the spontaneous and parent—infant inter-call interval lengths, respectively. (*b*) Average spontaneous (orange line) and parent–infant inter-call intervals (green line). The shaded regions represent the respective 95% confidence intervals. (*c*) Difference between spontaneous and parent–infant inter-call intervals (grey line). The grey shaded region represents the 95% confidence interval for the difference.

We now address the mechanism by which infants avoid overlapping their calls with their parents' calls. One possibility is that when an infant hears a parental call, it resets its call timing so that the interval between the parental call and infant response will be the same as the interval between the infant's own spontaneous calls (without parental calls in-between; figure 2*a*). Such resetting of the call timing would imply that the infant treats the calls it hears from its parents in the same way as its own calls. This would indicate that the infant does not have a well-developed self-monitoring capacity—it cannot distinguish its own call from another's. An alternative hypothesis is that when an infant hears a parental call, it responds within an interval that is shorter than the interval between spontaneous calls (figure 2*b*), similar to what happens between adult marmosets [22]. In this case, the infant is treating the calls produced by the parent differently from its own calls and is thus producing a vocal response, indicating the presence of self-monitoring. To test both hypotheses, we measured the average spontaneous and parent—infant call intervals. The spontaneous (orange) and parent–infant (green) inter-call intervals are statistically indistinguishable until P17 and then gradually become significantly distinct (figure 2*b*; 95% confidence intervals). To better visualize this developmental change, we plotted the difference between the call intervals (black line) and its 95% confidence interval (shaded grey; figure 2*c*). This result indicates that infant marmoset's self-monitoring capabilities develop gradually, allowing it to distinguish its own call from the calls produced by its parents.

### (c) Interactions between infants and parents are reciprocal

**Figure 3.**
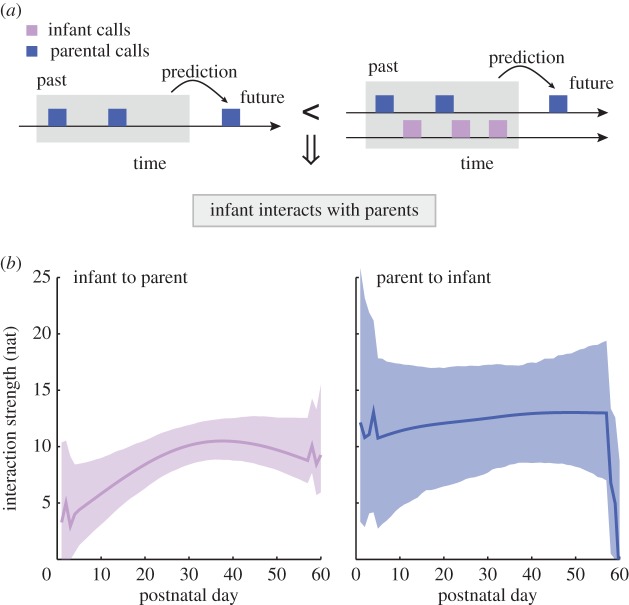
Granger causality quantifies interactions between infants and parents. (*a*) Illustration of the idea of Granger causality. An interaction from infant to parent vocalization exists if the prediction using only past parental vocalizations is worse than the prediction using the past parental and infant vocalizations. (*b*) Average interaction strength from infant to parent (purple line) and from parent to infant (blue line). Larger values indicate stronger interactions. Dashed regions indicate the respective 95% confidence intervals.

In order to establish vocal turn-taking and, more generally, social interaction, it is necessary that both infants and adults engage in vocal exchanges [1–3]. We used Granger causality to infer if the dynamics of infant vocalizations help predict the dynamics of parental vocalizations (or vice versa; figure 3*a*). In essence, we measured how well parental vocalizations can be predicted by considering both infant and parental vocalizations versus considering only the parent's own past vocal behaviour. Better prediction of parental vocalizations by including infant vocal behaviour would indicate that infants influence parents' vocal output. The same can be measured for parental influences on infant vocalizations. Better predictions result in larger absolute values of the interaction strength. Our data show that infants vocally interact with parents weakly in the first few days (not significantly different from zero; figure 3*b*, purple line) but gradually engage over the course of the month (*p* < 0.001, *t*-test for the slope of the robust regression). They then stabilize at a value similar to the interaction strength of their parents (*p* = 0.130, *t*-test for the slope of the robust regression). The parental effect is constant through development (*p* = 0.485, *t*-test for the coefficient of the robust regression). This result shows that infants gradually engage in vocal turn-taking during the first month of the postnatal period, but that parents are stable in their behaviour.

### (d) Infants change acoustic parameters of their calls following parental calls

**Figure 4.**
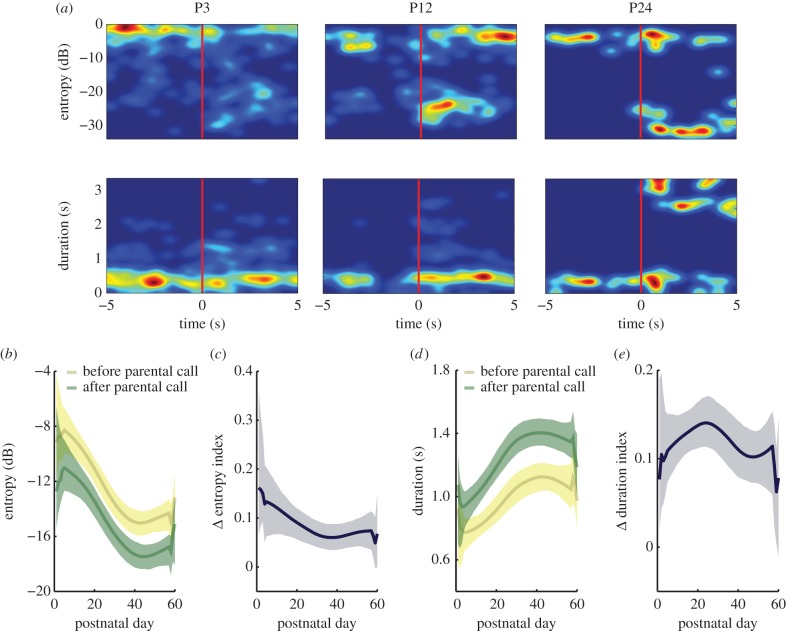
Infant call acoustics change after adult vocalizations. (*a*) Exemplar probability density distribution of entropy and duration for P3, P12 and P24 for a single marmoset. Warmer colours indicate higher probability density values. (*b*) Weighted average entropy of infant calls produced before adult call onset (yellow line) and after adult call offset (green line). The shaded regions indicate the respective 95% confidence intervals. (*c*) Index of entropy change (black line) ([entropy after − entropy before]/[entropy after + entropy before]). (*d*) Weighted average duration of infant calls produced before adult call onset (yellow line) and after adult call offset (green line). The shaded regions indicate the respective 95% confidence intervals. (*e*) Index of duration change (black line) ([duration after − duration before]/[duration after + duration before]).

We measured whether marmoset infants also change the acoustic characteristics of their calls following a vocal response from their parent. We calculated two main acoustic parameters: Wiener entropy and call duration [26,29]. Wiener entropy values that are close to zero indicate immature sound calls, while more negative values indicate more adult-like calls. More mature calls typically have lower entropies and longer durations [26]. For each infant and postnatal day, we calculated the distribution of entropy and duration of calls produced within 5 s before the parental call and 5 s after the parental call. The 5 s intervals before and after the parental call were chosen to match the approximately 10 s periodicity of call production in adult marmosets [22]. Using a probability distribution plot, figure 4*a* shows exemplars of the entropy and duration for P3, P12 and P24 of a single marmoset infant's calls. As the infant gets older, the entropy becomes lower and the duration becomes longer after the infant hears a parental call. For all infants together, we calculated the weighted average of the entropy and duration produced before the parental call onset and after parental call offset. Figure 4*b* shows that, throughout development, the entropy of calls produced after the parental call (green) is lower than the entropy of calls produced before the parental call (yellow). Figure 4*d* shows that call durations are longer after parental calls throughout development. To quantify these changes, we calculated entropy and duration change indices and their 95% confidence intervals (figure 4*c,e*). The positive entropy change index indicates that the entropy after the parental call is lower than the entropy before (i.e. the call is more adult-like in its spectral properties). The positive duration change index indicates that the duration of an infant call produced after a parental call is longer than one produced before. Figure 4*c,e* shows that the infant calls produced after a parental call are more adult-like, with lower entropy and longer duration.

**Figure 5.**
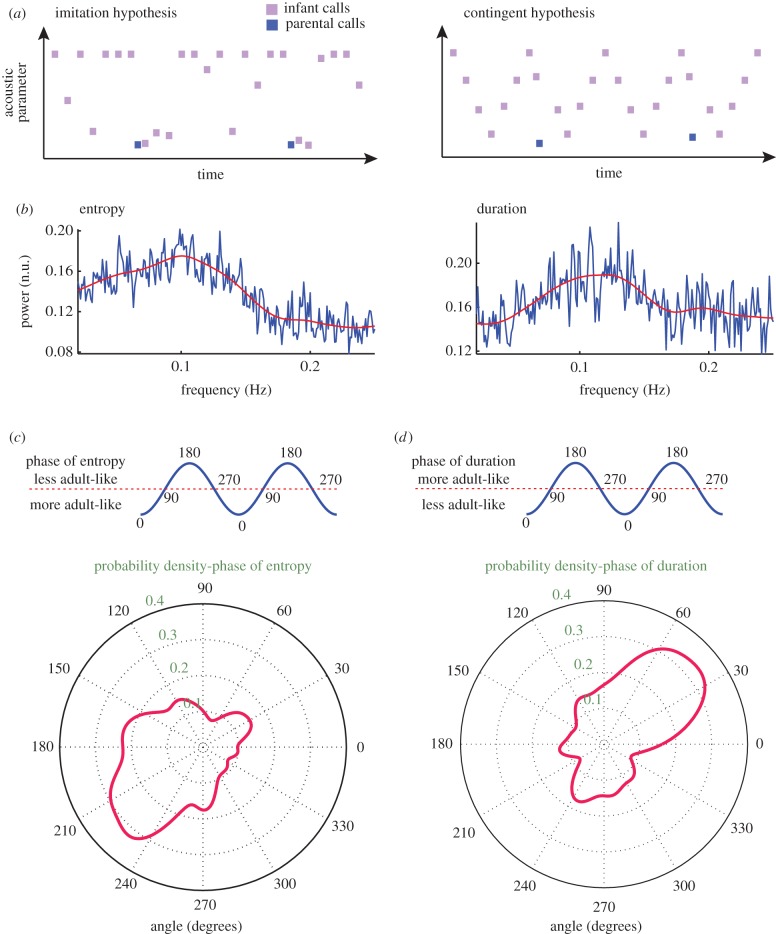
Imitation versus contingent hypotheses. (*a*) Schematic illustration of the imitation and contingent hypotheses. In the imitation hypothesis, irrespective of the infant vocalization dynamics, changes in infant call acoustics are solely driven by parental calls. In the contingent hypothesis, the dynamics of the infant call acoustics both modulates and is influenced by the dynamics of parental vocalization. (*b*) Power spectrum of the temporal dynamics of Wiener entropy and duration of infant calls. Powers were measured in normalized units (n.u.) (*c*) Probability density distribution of the phase of the infant call entropy dynamics at which the parents vocalize. More negative Wiener entropy values indicate more adult-like calls. The top panel is a representation of the phases. Zero and 180° represent the trough and peak, respectively. The bottom panel represents, on a polar coordinate (degrees), the probability density distribution of the most probable phases for each session. The solid red line indicates the values of the probability distribution. (*d*) Probability density distribution of the phase of the infant call duration dynamics at which the parents vocalize. Larger values indicate more adult-like calls. Convention is the same as in (*c*).

Two possible explanations exist as to why the infant calls are more adult-like after the infant hears a parental call. One possibility is that infants are trying to imitate parent calls, and the other possibility is that parental calls are contingent upon the dynamics of the acoustic change in infant calls (i.e. certain infant call features trigger parental responses). If the infant is trying to imitate the parental call, then the parental call should evoke more mature-sounding vocalizations, irrespective of the infant's vocal acoustics (figure 5*a*). If the parents are producing vocalizations contingently to the dynamics of acoustic features in infant calls, then the infant and parental calls should have a temporal relationship. Such a dynamic relationship could be established if two features were present: (i) acoustic changes in infant calls exhibit a temporal regularity; and (ii) the parental calls entrain to a specific phase of this regularity (figure 5*a*). To test both hypotheses, we calculated the power spectrum of the acoustic features of infant calls and the phase at which the parents produced their vocalizations. Consistent with the contingent call hypothesis, the Wiener entropy and duration of infant calls showed a periodicity at approximately 0.1 Hz (figure 5*b*), and there was a clear concentration of parental responses at the phase of approximately 230° for entropy changes (figure 5*c*). Thus, parents tend to produce their vocal responses when they hear infant calls that are at the transition point from less to more adult-like (i.e. from high entropy calls to low entropy calls). This is consistent with the result in figure 4*b*, where the infants produce more adult-like calls after hearing parental calls. We also observed that the parental calls were produced at approximately 45° of the phase of infant call durations (figure 5*d*), i.e. between the transitions from short-duration to longer-duration calls. This is consistent with figure 4*d*, where the infants produce longer adult-like calls after listening to parental calls.

### (e) Does parental feedback influence the rate of infant turn-taking development?

**Figure 6.**
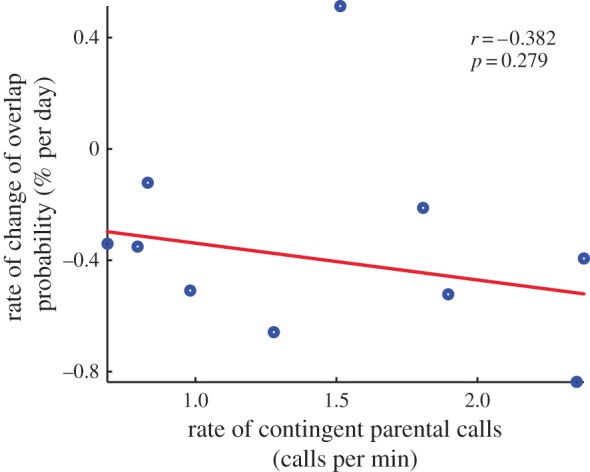
Relationship between rate of contingent parental calls and rate of change in the probability to overlap. Each dot represents the data point for each infant. Red line is the linear regression line fitted to the data using robust linear regression. (Online version in colour.)

While contingent parental responsiveness may shape the acoustic structure of infant calls in both human infants [13,30,31] and marmoset infants [26], there is no evidence in human infants that the rate of vocal turn-taking development is facilitated by parental responsiveness. Yet Miller and colleagues recently claimed that juvenile marmosets learn how to take turns from their parents [32]. Their study, however, did not directly test the role of parental feedback on infant vocal turn-taking behaviour. To do so, one must compare the rate of contingent parental calls with how quickly each infant decreases the number of their overlapping calls. We tested this hypothesis using marmoset infants at the correct life-history stage for comparison with humans and found no evidence that the rate of contingent calls by parents influences the development of vocal turn-taking (figure 6; Spearman *ρ* = −0.382, *p*-value = 0.279).

## 3. Discussion

Vocal turn-taking is a ubiquitous form of social interaction in our lives. It is a communication system that exhibits the properties of a dynamical system: two individuals become coupled to each other via acoustic exchanges, mutually affect each other (e.g. phase locking and entrainment), and thereby produce an autonomous, self-sustaining organization [1–4]. In humans, vocal turn-taking develops during the first year of life [7–11]. In the first postnatal months, infants frequently overlap their vocalizations with their parents' utterances. By approximately nine months of age, they can engage in proto-conversations with adult-like turn-taking dynamics. In this study, we investigated the development of vocal turn-taking in marmoset monkeys. Turn-taking in adult marmosets has the same universal features of human conversational turn-taking (albeit on a different timescale) and exhibits the essential features of a coupled oscillator system [22].

We tested marmoset infants in a directed context in which they could vocally interact with their parents, starting on postnatal day 1. We sampled densely thereafter until they were two months of age. Because marmoset monkeys develop 12 times faster than humans [27,28], this time interval represents the equivalent of the first 2 years of human postnatal life. We found that early in postnatal life, marmoset infants overlap their vocalizations with their parents about 25% of the time, an overlap probability consistent with what is observed in human 3-month-olds [9]. This overlap probability decreases to about 15% in marmoset infants by the time they are two months of age, also consistent with what is observed in the near age-equivalent human infant (18-month-olds; [9]). Adult marmosets rarely overlap their vocalizations [22]. Thus, the fact that turn-taking is still developing at two months of age in marmosets is analogous to the way that human infants at 18 months of age still exhibit immature vocal interaction dynamics. We also showed that the amount of observed vocalization overlap is larger than what is expected by chance early in postnatal life, similar to what was observed in 13–15-week-old human infants [33]. Thus, marmoset infants undergo the same developmental trajectory for vocal turn-taking as humans, and do so during the same life-history stage.

During the changes in vocal turn-taking by infants, it is important to know whether or not infants, parents or both are changing their interactive behaviour. Using Granger causality measures, we found that infants only weakly interact with parents in the first postnatal week but gradually engage more over the course of first month. Parents, however, do not change how they interact with their infant; their vocal behaviour remains steady over time. This, too, is consistent with what is observed in human infant–parent vocal interactions [9]. Mechanistically, our data suggest that the lack of robust interactions with parents early in life may be because the neural system of self-monitoring is not fully developed [34]. We tested this by measuring the interval between the infants' spontaneous calls and the interval between parent and infant calls. In the first two postnatal weeks, the intervals between spontaneous infant calls and parent–infant calls were statistically indistinguishable; infants seem unable to distinguish their own calls from their parents' calls at this early stage. Thus, infant marmosets' self-monitoring capabilities develop in the first months of postnatal life, allowing the infants to better participate in vocal turn-taking. That vocal self-monitoring must develop is consistent with data from a study of speech self-regulation in human children [35].

The system of vocal turn-taking between infants and parents is one in which parental responses are contingent upon infant vocalizations. This provides an opportunity for the infant to learn from the parents how to take turns during a vocal exchange. Although it is an intriguing possibility, as far as we know, there is no evidence that parental feedback influences infant turn-taking behaviour in humans. However, such an influence was recently claimed to occur in 4–12-month-old juvenile marmoset monkeys (age-equivalent of a 4–12-year-old human) [32]. The claim was based on the finding that parents responded differently to juvenile calls that overlapped parental calls compared to calls that did not overlap. This result was interpreted as evidence that parents were giving reinforcement signals to the juvenile marmosets when the juveniles produced calls without overlaps [32]. If this hypothesis was correct, the amount of contingent parental responses should be correlated with the rate of turn-taking development, so that infants with more responsive parents will learn to turn take faster. We directly tested this possibility of parental influences on turn-taking development and found no relationship between the maturation rate of vocal turn-taking and overall frequency of contingent parental responses. Another possibility for learning in this infant–parent vocal system is that contingent parental responses influence infant vocal acoustics. Studies of naturalistic human infant–parent interactions [12–15], as well as experimental studies [30,31], reveal that contingent parental responses influence the acoustic structure of subsequent infant vocalizations, making them sound more mature (i.e. speech-like). Along similar lines, the maturation rate of marmoset infant vocalizations is influenced by contingent feedback from parents [26]. The real-time basis for that influence was revealed in the current study: Subsequent to their parents' vocalizations, marmoset infants increasingly produce longer and more tonal (low entropy) calls over the course of development. This is yet another parallel with human vocal turn-taking development, and consistent with the many ways infants can learn from parents beyond imitation [36].

The similarities between the developmental trajectories of vocal turn-taking in humans and marmoset monkeys are striking both in their form and timing (after accounting for the relative rapidity of marmoset development compared to humans). Based on these findings, what can we conclude with regard to how such a developmental system for vocal turn-taking evolved [16,17]? Typically, with any behaviour that two closely related species share, it can be inferred that their last common ancestor also exhibited that behaviour. Marmoset monkeys are not very closely related to humans, especially when compared to Old World primates, like chimpanzees or macaque monkeys. If there was evidence that these other primates exhibited vocal turn-taking, then one could conclude that the ancestral species to both marmosets (and other New World monkeys) and Old World primates (including humans) had a turn-taking capacity. However, there is no such evidence to date. Despite suggestions to the contrary [17], call-and-response behaviours are not the same as turn-taking; they do not exhibit the ‘coupled’ nature of true social interactions observed in marmosets [22], and in human interactions more generally [1–4]. Thus, we conclude that vocal turn-taking by marmosets and humans is an instance of convergent evolution, possibly as a result of pressures on both species to adopt a cooperative breeding strategy, and perhaps through the activation of a shared (homologous) neuronal network [16,37].

Cooperative breeding is a prosocial behaviour found only in approximately 3% of mammals [38]. Among primates, only humans and callitrichids (the primate taxon that includes marmosets) are known to exhibit this strategy [37,38]. Cooperative breeding occurs when the rearing of infants is greatly reliant on a concerted effort among the breeding female, breeding male, non-breeding siblings and occasionally other familiar but unrelated group members [38,39]. In contrast to other monkeys, marmoset carers actively and frequently provision food for offspring, and compete with each other for the opportunity to carry offspring [39–42]. This cooperative breeding framework, in which non-parents within a social group spontaneously care for offspring other than their own, has been argued to drive human cognition [37]. Vocal turn-taking and its development may be a specific instance within the suite of prosocial behaviours exhibited by humans and marmosets.

## 4. Material and methods

### (a) Subjects

The data analysed in this work is a subset of the dataset that was previously published [26]. The subjects used in the study were 10 infants and 6 adults (3 male–female pairs, more than 2 years old), captive common marmosets (*Callithrix jacchus*) housed at Princeton University. The colony room is maintained at a temperature of approximately 27°C and 50–60% relative humidity, with a 12 L : 12 D light cycle. Marmosets live in family groups; all were born in captivity. They had *ad libitum* access to water and were fed daily with standard commercial chow supplemented with fruits and vegetables. Additional treats (peanuts, cereal, dried fruits and marshmallows) were used prior to each session to transfer the animals from their home-cage into a transfer cage.

### (b) Experimental set-up

Beginning on their first postnatal day, we recorded the vocalizations of marmoset monkey infants in two different contexts: undirected (i.e. social isolation) and directed (with auditory, but not visual, contact with their mother or father). Only the directed context data were used in this work. The details of the full experiment are described in Takahashi *et al.* [26]. Here, we will describe only the directed-context experiment. Early in life, parents always carry infants. Thus, the parent carrying the infant(s) was first lured from the home cage into a transfer cage using treats. The infant marmoset was then gently separated from the adult and taken to the experiment room where it was placed in a second transfer cage on a flat piece of foam. The adult was brought to the room after the infant. During this set-up procedure and throughout the experiment, an opaque curtain prevented the infant and the parent from having visual contact. The directed experiment lasted for 10–15 min. For the data analysis, we used the first 10 min of recording for each session. The order of which parent participated in the interaction was counterbalanced. The vocalizations we observed were identical in type to those produced during natural separation from parents (e.g. when parents push them off or when they transfer them to the other parent for carrying or feeding). The cage rested on a table (0.66 m in height) in one of two opposing corners of the room. The testing corner was counterbalanced across sessions. A speaker was placed at a third corner equidistant from both testing corners and pink noise (amplitude decaying inversely proportional to frequency) was broadcast at approximately 45 dB (at 0.88 m from the speaker) in order to mask occasional noises produced outside the testing room. A digital recorder (ZOOM H4n Handy Recorder) was placed directly in front of the transfer cages at a distance of 0.76 m. Audio signals were acquired at a sampling frequency of 96 kHz. The order of the infant testing was also counterbalanced, so that if two infants were recorded in the same day, the order in which the infants were recorded next time was switched. A second digital recorder (ZOOM H4n Handy Recorder) was placed directly in front of the parent at a distance of 0.76 m from the transfer cage. For this work, we included data recorded until postnatal day 60. The number of directed experiments for each infant was 17, 13, 13, 18, 24, 24, 20, 20, 21, 22 (192 sessions). The mean number of recorded sessions per week was 22.375 ± 1.845 (mean ± s.d.).

### (c) Detection of syllables

To determine the onset and offset of a syllable, a custom-made MATLAB^®^ routine automatically detected the onset and offset of any signal that differed from the background noise in a specific frequency range. To detect the differences, we first bandpass filtered the entire recording signal between 6 and 10 kHz. This corresponds to the dominant frequency of marmoset calls, i.e. the frequency with the highest power, which is not necessarily the fundamental frequency (F0), i.e. the lowest frequency of the periodic components of the sound. Second, we resampled the signal to 1 kHz sampling rate, applied the Hilbert transform and calculated the absolute value to obtain the amplitude envelope of the signal. The amplitude envelope was further low-pass filtered to 50 Hz. A segment of the recording without any vocalizations (silent) was chosen as a comparison baseline. The 99th percentile of the amplitude value in the silent period was used as the detection threshold. Sounds with an amplitude envelope higher than the threshold were considered a possible vocalization. Finally, to ensure that sounds other than call syllables were not included, a researcher verified whether each detected sound was a vocalization or not, based on the spectrogram. To distinguish the identity of the caller for each vocalization, we used the difference in sound level of the same call recorded in both microphones.

### (d) Quantification of acoustic parameters

After detecting the onset and offset of syllables, a custom-made MATLAB^®^ routine calculated the duration and Wiener entropy of each syllable. The duration of a syllable is the difference between the offset and onset time of the vocalization detected by our custom-made program. The Wiener entropy is the logarithm of the ratio between the geometric and arithmetic means of the values of the power spectrum for different frequencies [29]. The Wiener entropy is a non-positive number and represents the broadband properties of the signal's power spectrum. The closer the signal is to white noise, the higher (closer to zero) the Wiener entropy value.

### (e) Classification of type of syllables

Each automatically detected syllable was manually classified as phee, phee-cry, subharmonic-phee, cry, twitter, trill and trill-phee based on the spectro-temporal profile measured by the spectrogram. To ensure the validity of our classification procedure, 10 sessions chosen at random were classified by two different individuals and compared. The classification matched in more than 99.9% of the calls. The seven call types show very distinct spectro-temporal profiles and can be easily classified by eye [43,44]. Briefly, the phee is a tone-like, long call with F0 at around 7–10 kHz; the twitter is a short, upward FM sweep; the trill is defined by sinusoidal-like FM throughout the entire call; the cry is a broad-band call, with F0 around 600 Hz; the phee-cry is a combination of the phee and cry in any order, with each component lasting at least 50 ms; the trill-phee is a combination of the trill and phee in any order, with each component lasting at least 50 ms. A subharmonic-phee is similar to a phee, but has a strong harmonic component around 3.5–5 kHz. We classified a call as a subharmonic-phee if the harmonic component around 3.5–5 kHz was visible in the spectrogram for at least 50 ms. We defined a whole (i.e. multisyllabic) call as any uninterrupted sequence of utterances of the same type separated (previous offset to next onset) by less than 500 ms for infant calls [26] and less than 1000 ms for adult calls [22]. The acoustic parameter of a whole call is the average of the acoustic parameters of each syllable in the call.

### (f) Observed and expected probability to overlap

An infant call was considered to overlap an adult call if the onset of the infant call was between the onset and offset of the adult call. The probability that infant calls overlapped parental calls was calculated as the ratio between the number of infant calls overlapping parental calls divided by the total number of parental calls in a session. To verify whether the decrease in the proportion of overlapped calls was significant, we fitted a robust linear regression to the probability that infant calls overlapped parental calls during development (MATLAB^®^ robustfit). The robust linear regression was used because it is more robust against deviation of the assumptions necessary for the validity of ordinary linear regression like the homoscedasticity of the data. A cubic spline was fitted to the overlap probability of each infant using the MATLAB^®^ csaps function. The average of the curves for all infants represents the population average curve. We used a bootstrap method to calculate a 95% confidence interval for the population average curve. We calculated the expected probability to overlap by chance using a permutation procedure. For each session, the duration and interval between calls for the infant and adult was randomly resampled with replacement, and a permuted sequence was built. Then the probability for the infant call to overlap an adult call was calculated for the permuted sequences. A cubic spline was fitted using csaps, and the population average was calculated. We repeated this procedure 1000 times and calculated the 2.5, 50 and 97.5 percentiles. The 50 percentile curve represented the expected probability to overlap. To obtain the strength of overlap avoidance, we calculated the difference between the observed and expected probability to overlap, and divided by the expected probability. The confidence interval was calculated using the bootstrap procedure. The significance region at 5% was obtained by calculating the difference between the median and the 2.5 and 97.5 percentile values for expected probability to overlap.

### (g) Spontaneous and alternating inter-call intervals

We calculated the spontaneous inter-call interval for infant calls, which is the interval between the onset of an infant call and the offset of the preceding infant call, without an interruption by an adult call. We calculated the average for each session and then fitted a cubic spline using MATLAB^®^ csaps for each infant. The average of the curves represented the population values. We used a bootstrap method to calculate a 95% confidence interval for the population average curves. We also calculated the parent–infant inter-call interval, which is the interval between the offset of an infant call and the onset of the preceding adult call without an interruption from another call. The population curve and the 95% confidence intervals were obtained in the same way as for the spontaneous inter-call intervals. The difference between spontaneous and parent–infant inter-call intervals was calculated using the difference between the respective cubic spline fits. The 95% confidence interval was calculated using a bootstrap procedure.

### (h) Granger causality

We calculated the Granger causality between the onsets of syllables. Because this method allows us to model the dynamical properties of call production, naturally taking into account the relation between syllables and calls, it was not necessary to combine syllables. The result using the offset of syllables was essentially the same. We adapted a Granger causality method that was initially developed to make inferences about the interaction patterns between neurons [45]. Briefly, we fitted a generalized linear model to model the dynamics of the onset of syllables. To test whether infants influence parental vocal dynamics, we compared two models: one in which the only predictor is the past onset times of parental vocalizations and one that considers the past onset times of both parental and infant vocalizations. If the model accounting for both parental and infant vocalizations is a better fit, we can infer that infants significantly contribute to vocal interactions with their parents. The difference in the maximum likelihood of both models reveals the strength of interaction [45]. We inferred the interaction strength of parent to infant in the same way. We calculated the strength of the interaction for each session and then fitted a cubic spline using MATLAB^®^ csaps for each infant. The average of the curves represented the population values. We used a bootstrap method to calculate a 95% confidence interval for the population average curves. We were interested in the absolute value of the strength of the interaction, so we calculated the absolute value of those estimates. We fitted a robust linear regression for the interaction strength from infant to parent between 1–30 postnatal days and 31–60 postnatal days. We also fitted a robust linear regression for the interaction strength from parent to infant for all sessions.

### (i) Acoustic parameter of infant calls before and after parental call

We used kde2d (MATLAB^®^ file exchange #17204 by Zdravko Botev) to calculate the probability density of the Wiener entropy and duration of infant calls produced before the onset and after the offset of adult calls. To calculate the weighted average of the entropy of infant calls produced before the onset and after the offset of parental calls, we calculated the sum of the entropy weighted by the probability of occurrence at each time point before the onset and after the offset for each session. Only sessions where more than four parental calls were produced were included in the analysis to allow reliable estimation of the probability of infant call occurrence (188 sessions). Then we calculated the entropy change index, which is defined as the difference between the averages divided by the sum of the averages. For each infant, we used MATLAB^®^ csaps to fit a cubic spline curve to the weighted average of entropy before the onset and after the offset of parental calls, and to the entropy change index. The 95% confidence intervals were obtained by a bootstrap procedure. We calculated analogously the weighted average and change index curves for the duration of calls.

### (j) Power spectrum of infant call acoustics

To verify whether the Wiener entropy and the duration of infant calls exhibit intrinsic rhythmicity, we calculated, for each session, the power spectrum of the sequence of entropy and duration of infant calls. Because the calls are irregularly spaced, we used the Lomb–Scargle spectrogram to calculate the power spectrum of the sequence of Wiener entropy and duration of the calls (fastlomb, MATLAB^®^ file exchange #22215 by Christos Saragiotis). We normalized the power spectrum for each session by dividing the power spectrum by its maximum value, so that the peak value is 1. We averaged the power spectrum for all sessions. To better visualize the shape of the power spectrum, we also fitted a cubic spline to the average power spectrum using csaps.

### (k) Phase of the parental call

To calculate the phase of the infant vocalization dynamics at which the parents vocalized, for each session we first interpolated the sequence of Wiener entropy and duration of infant calls. We used a cubic spline interpolation using MATLAB^®^ csaps. Then we applied the Hilbert transform to the interpolated sequences and obtained the angle of the transformation at the time point where the parent vocalized. For each session, we calculated the probability distribution of the phases (with respect to the Wiener entropy and duration sequences) at which the parents vocalized. Then we determined the mode (peak) of the probability distribution to represent the preferred phase for each session. To represent the population preference, we plotted the circular probability density of the preferred phases parental call production of all sessions and animals. We used circ_ksdensity2 to calculate the circular probability density (MATLAB^®^ file exchange #44072 by Dylan Muir).

### (l) Correlation between rate of contingent calls and rate of change of overlap probability

A parental call was classified as contingent response to an infant call if the onset of parental call was separated by less than 5 s from the offset of the infant call and there is no other call between both calls. For each marmoset, the rate of contingent calls was the average of the number of contingent calls divided by the duration of the sessions. To calculate the rate of change of overlap probability, for each infant we first fitted a cubic spline to the infant probability of overlapping an adult call. Then we calculated the average slope of the fitted curve. To test whether the rate of contingent calls was correlated with the rate of change of overlap probability, we used a Spearman correlation. We fitted a robust linear regression between the rate of contingent call and the rate of change of overlap probability.

## References

[1] Dumas G, de Guzman GC, Tognoli E, Kelso JS 2014 The human dynamic clamp as a paradigm for social interaction. Proc. Natl Acad. Sci. USA 111, E3726–E3734. (10.1073/pnas.1407486111) 25114256 PMC4156776

[2] Oullier O, de Guzman GC, Jantzen KJ, Lagarde J, Kelso JAS 2008 Social coordination dynamics: measuring human bonding. Soc. Neurosci. 3, 178–192. (10.1080/17470910701563392) 18552971 PMC2156197

[3] De Jaegher H, Di Paolo E, Gallagher S 2010 Can social interaction constitute social cognition? Trends Cogn. Sci. 14, 441–447. (10.1016/j.tics.2010.06.009) 20674467

[4] Fogel A, Garvey A 2007 Alive communication. Infant Behav. Dev. 30, 251–257. (10.1016/j.infbeh.2007.02.007) 17376534

[5] Sacks H, Shegloff EA, Jefferson G 1974 Simplest systematics for organization of turn-taking for conversation. Language 50, 696–735. (10.2307/412243)

[6] Stivers T et al. 2009 Universals and cultural variation in turn-taking in conversation. Proc. Natl Acad. Sci. USA 106, 10 587–10 592. (10.1073/pnas.0903616106) 19553212 PMC2705608

[7] Stern DN, Jaffe J, Beebe B, Bennett SL 1975 Vocalizing in unison and in alternation: two modes of communication within the mother–infant dyad. Ann. NY Acad. Sci. 263, 89–100. (10.1111/j.1749-6632.1975.tb41574.x) 1060437

[8] Elias G, Broerse J 1996 Developmental changes in the incidence and likelihood of simultaneous talk during the first two years: a question of function. J. Child Lang. 23, 201–217. (10.1017/S0305000900010151) 8733567

[9] Hilbrink EE, Gattis M, Levinson SC 2015 Early developmental changes in the timing of turn-taking: a longitudinal study of mother-infant interaction. Front. Psychol. 6, 1492 (10.3389/fpsyg.2015.01492) 26483741 PMC4586330

[10] Jasnow M, Feldstein S 1986 Adult-like temporal characteristics of mother–infant vocal interactions. Child Dev. 57, 754–761. (10.2307/1130352) 3720401

[11] Kajikawa S, Amano S, Kondo T 2004 Speech overlap in Japanese mother–child conversations. J. Child Lang. 31, 215–230. (10.1017/S0305000903005968) 15053091

[12] Bloom K, Russell A, Wassenberg K 1987 Turn taking affects the quality of infant vocalizations. J. Child Lang. 14, 211–227. (10.1017/S0305000900012897) 3611239

[13] Gros-Louis J, West MJ, King AP 2014 Maternal responsiveness and the development of directed vocalizing in social interactions. Infancy 19, 385–408. (10.1111/infa.12054)

[14] Masataka N 1993 Effects of contingent and noncontingent maternal stimulation on the vocal behaviour of three- to five-month old Japanese infants. J. Child Lang. 20, 303–312. (10.1017/S0305000900008291) 8376471

[15] Hsu H-C, Fogel A 2001 Infant vocal development in a dynamic mother–infant communication system. Infancy 2, 87–109. (10.1207/S15327078IN0201_6) 33451227

[16] Borjon JI, Ghazanfar AA 2014 Convergent evolution of vocal cooperation without convergent evolution of brain size. Brain Behav. Evol. 84, 93–102. (10.1159/000365346) 25247613 PMC7592170

[17] Levinson SC 2016 Turn-taking in human communication—origins and implications for language processing. Trends Cogn. Sci. 20, 6–14. (10.1016/j.tics.2015.10.010) 26651245

[18] Deacon TW 1990 Rethinking mammalian brain evolution. Am. Zool. 30, 629–705. (10.1093/icb/30.3.629)

[19] Finlay BL, Darlington RB, Nicastro N 2001 Developmental structure of brain evolution. Behav. Brain Sci. 24, 263–308. (10.1017/S0140525X01003958) 11530543

[20] Schneirla TC 1949 Levels in the psychological capacities of animals. In Philosophy for the future (eds Sellars RW, McGill VJ, Farber M), pp. 243–286. New York, NY: Macmillan.

[21] Egnor SER, Hauser MD 2004 A paradox in the evolution of primate vocal learning. Trends Neurosci. 27, 649–654. (10.1016/j.tins.2004.08.009) 15474164

[22] Takahashi DY, Narayanan DZ, Ghazanfar AA 2013 Coupled oscillator dynamics of vocal turn-taking in monkeys. Curr. Biol. 23, 2162–2168. (10.1016/j.cub.2013.09.005) 24139740

[23] Ghazanfar AA, Takahashi DY 2014 The evolution of speech: vision, rhythm, cooperation. Trends Cogn. Sci. 18, 543–553. (10.1016/j.tics.2014.06.004) 25048821 PMC4177957

[24] Choi JY, Takahashi DY, Ghazanfar AA 2015 Cooperative vocal control in marmoset monkeys via vocal feedback. J. Neurophysiol. 114, 274–283. (10.1152/jn.00228.2015) 25925323 PMC4507967

[25] Elowson AM, Snowdon CT, Lazaro-Perea C 1998 Infant ‘babbling’ in a nonhuman primate: complex vocal sequences with repeated call types. Behaviour 135, 643–664. (10.1163/156853998792897905)

[26] Takahashi DY, Fenley AR, Teramoto Y, Narayanan DZ, Borjon JI, Holmes P, Ghazanfar AA 2015 The developmental dynamics of marmoset monkey vocal production. Science 349, 734–738. (10.1126/science.aab1058) 26273055

[27] de Castro Leão A, Doriá Neto AD, Cordeiro de Sousa MB 2009 New developmental stages for common marmosets (*Callithrix jacchus*) using mass and age variables obtained by K-means algorithm and self-organizing maps (SOM). Comp. Biol. Med. 39, 853–859. (10.1016/j.compbiomed.2009.05.009) 19651403

[28] Schultz-Darken N, Braun KM, Emborg ME 2015 Neurobehavioral development of common marmoset monkeys. Dev. Psychobiol. 58, 141–158. (10.1002/dev.21360) 26502294 PMC4829073

[29] Tchernichovski O, Mitra PP, Lints T, Nottebohm F 2001 Dynamics of the vocal imitation process: how a zebra finch learns its song. Science 291, 2564–2569. (10.1126/science.1058522) 11283361

[30] Goldstein MH, King AP, West MJ 2003 Social interaction shapes babbling: testing parallels between birdsong and speech. Proc. Natl Acad. Sci. USA 100, 8030–8035. (10.1073/Pnas.1332441100) 12808137 PMC164707

[31] Goldstein MH, Schwade JA 2008 Social feedback to infants’ babbling facilitates rapid phonological learning. Psychol. Sci. 19, 515–523. (10.1111/J.1467-9280.2008.02117.X) 18466414

[32] Chow CP, Mitchell JF, Miller CT 2015 Vocal turn-taking in a non-human primate is learned during ontogeny. Proc. R. Soc. B 282, 20150069 (10.1098/rspb.2015.0069) PMC4424641 25904663

[33] Elias G, Hayes A, Broerse J 1986 Maternal control of co-vocalization and inter-speaker silences in mother–infant vocal engagement. J. Child Psychol. Psychiatry 27, 409–415. (10.1111/j.1469-7610.1986.tb01842.x) 3733920

[34] Takahashi DY, Narayanan D, Ghazanfar AA 2012 A computational model for vocal exchange dynamics and their development in marmoset monkeys. IEEE International Conference on Development and Learning and Epigenetic Robotics (ICDL) 2012, San Diego, CA, 7–9 Nov, pp. 1–2. San Diego, CA: IEEE. (10.1109/DevLrn.2012.6400844)

[35] MacDonald EN, Johnson EK, Forsythe J, Plante P, Munhall KG 2012 Children's development of self-regulation in speech production. Curr. Biol. 22, 113–117. (10.1016/j.cub.2011.11.052) 22197241 PMC3267900

[36] Tchernichovski O, Marcus G 2014 Vocal learning beyond imitation: mechanisms of adaptive vocal development in songbirds and human infants. Curr. Opin. Neurobiol. 28, 42–47. (10.1016/j.conb.2014.06.002) 25005823 PMC4177410

[37] Burkart JM, Hrdy SB, van Schaik CP 2009 Cooperative breeding and human cognitive evolution. Evol. Anthropol. 18, 175–186. (10.1002/evan.20222)

[38] Hrdy SB 2005 Evolutionary context of human development: the cooperative breeding model. In Attachment and bonding: a new synthesis; from the 92nd Dahlem Workshop Report (eds Carter C, Ahnert L, Grossmann K, Hrdy S, Lamb M, Porges S, Sachser N), pp. 9–32. Cambridge, UK: MIT Press.

[39] Burkart JM, van Schaik CP 2010 Cognitive consequences of cooperative breeding in primates? Anim. Cogn. 13, 1–19. (10.1007/s10071-009-0263-7) 19629551

[40] Yamamoto ME, de Araújo Lopes F 2004 Effect of removal from the family group on feeding behavior by captive *Callithrix jacchus*. Int. J. Primatol. 25, 489–500. (10.1023/B:IJOP.0000019164.98756.9c)

[41] Santos CV, French JA, Otta E 1997 Infant carrying behavior in callitrichid primates: *Callithrix* and *Leontopithecus*. Int. J. Primatol. 18, 889–907. (10.1023/A:1026340028851)

[42] Snowdon CT, Cronin KA 2007 Cooperative breeders do cooperate. Behav. Process. 76, 138–141. (10.1016/j.beproc.2007.01.016) PMC2080785 17703900

[43] Bezerra BM, Souto A 2008 Structure and usage of the vocal repertoire of *Callithrix jacchus*. Int. J. Primatol. 29, 671–701. (10.1007/s10764-008-9250-0)

[44] Pistorio AL, Vintch B, Wang X 2006 Acoustic analysis of vocal development in a New World primate, the common marmoset (*Callithrix jacchus*). J. Acoust. Soc. Am. 120, 1655–1670. (10.1121/1.2225899) 17004487

[45] Kim S, Putrino D, Ghosh S, Brown EN 2011 A Granger causality measure for point process models of ensemble neural spiking activity. PLoS Comput. Biol. 7, e1001110 (10.1371/journal.pcbi.1001110) 21455283 PMC3063721

